# Effects of a dietary intervention with lacto-ovo-vegetarian and Mediterranean diets on apolipoproteins and inflammatory cytokines: results from the CARDIVEG study

**DOI:** 10.1186/s12986-023-00773-w

**Published:** 2024-02-01

**Authors:** Giuditta Pagliai, Marta Tristan Asensi, Monica Dinu, Francesca Cesari, Alessia Bertelli, Anna Maria Gori, Betti Giusti, Rossella Marcucci, Francesco Sofi, Barbara Colombini

**Affiliations:** 1 Department of Experimental and Clinical Medicine, School of Human Health Sciences, University of Florence, Florence, Italy; 2 Atherotrombotic Diseases Unit, Careggi University Hospital, Florence, Italy; 3 Unit of Clinical Nutrition, Careggi University Hospital, Florence, Italy

**Keywords:** Cardiovascular risk; apolipoproteins; Mediterranean diet; vegetarian diet

## Abstract

**Background:**

Apolipoproteins have been recently proposed as novel markers of cardiovascular disease (CVD) risk. However, evidence regarding effects of diet on apolipoproteins is limited.

**Aim:**

To compare the effects of Mediterranean diet (MD) and lacto-ovo vegetarian diet (VD) on apolipoproteins and traditional CVD risk factors in participants with low-to-moderate CVD risk.

**Methods:**

Fifty-two participants (39 women; 49.1 ± 12.4 years), followed MD and VD for 3 months each. Medical and dietary information was collected at the baseline. Anthropometric parameters and blood samples were obtained at the beginning and the end of interventions.

**Results:**

MD and VD resulted in significant improvement in anthropometric and lipid profiles. Both diets led to a reduction in most of the inflammatory parameters. As for apolipoproteins, a significant change was observed for ApoC-I after VD (+ 24.4%; *p* = 0.020). MD led to a negative correlation between ApoC-III and carbohydrates (R = − 0.29; *p* = 0.039) whereas VD between ApoD and saturated fats (R = − 0.38; *p* = 0.006). A positive correlation emerged after VD between HDL and ApoD (R = 0.33; *p* = 0.017) and after MD between plasma triglycerides and ApoC-I (R = 0.32; *p* = 0.020) and ApoD (R = 0.30; *p* = 0.031). IL-17 resulted to be positively correlated with ApoB after MD (R = 0.31; *p* = 0.028) and with ApoC-III after VD (R = 0.32; *p* = 0.019). Subgroup analysis revealed positive effects on apolipoproteins from both diets, especially in women, individuals older than 50 years-old or with < 3 CVD risk factors.

**Conclusions:**

Both diets seem to improve CVD risk, however, MD showed a greater positive effect on apolipoproteins in some subgroups, thus suggesting how diet may influence new potential markers of CVD risk.

*Trial registration*: registered at clinicaltrials.gov (identifier: NCT02641834) on December 2015.

**Supplementary Information:**

The online version contains supplementary material available at 10.1186/s12986-023-00773-w.

## Background

Cardiovascular disease (CVD) remains the leading cause of mortality worldwide and causes an estimated 18 million deaths per year [1]. An altered lipid profile is one of the most widely recognized risk factors for CVD, which together with chronic low-grade inflammation favors the formation and progression of atherosclerosis plaque. However, in recent years attention has focused on the role that apolipoproteins might play as markers of CVD since apolipoproteins are important structural components of circulating lipoproteins that may influence atherosclerotic plaque progression by regulating lipoprotein metabolism. ApoB is probably the most studied and recognized marker of cardiovascular risk, given its presence in lipoproteins associated with increased risk, such as VLDL, IDL and LDL. In addition, ApoA-I—which is mainly found in HDL lipoprotein—is related to an inverse risk of CVD due to its role in reverse cholesterol transport. Furthermore, it has been suggested that the ApoB/ApoA-I ratio is a better risk marker than the total cholesterol/HDL cholesterol ratio in both sexes and in all age groups [2–4]). ApoC-III appears to play a key role in the pathophysiology of atherosclerosis, as it is related to the metabolism of triglyceride-rich lipoproteins, with elevated ApoC-III levels being associated with increased levels of hypertriglyceridemia [5]. However, scientific evidence on other apolipoproteins, such as ApoE, ApoD and ApoC-I and the possible role in lipid metabolism and their relationship with atherosclerotic risk still remains scarce and controversial.

Robust evidence supports that healthy dietary patterns based on high consumption of plant-based foods such as vegetables, whole grains, legumes, nuts, seeds and fruits, reduce cardiovascular risk [6, 7]. Both Mediterranean diet (MD) and lacto-ovo vegetarian diet (VD) seem to have a beneficial effect on the inflammatory profile by improving several pro-inflammatory markers [8, 9] as well as on the lipid profile, MD by improving triglyceride levels while VD improving LDL and total cholesterol [10]. Given the relationship between lipid profile and the level of circulating apolipoproteins, there is growing interest in the possible role of diet in influencing cardiovascular risk through the modulation of apolipoproteins.

Some studies suggest that the MD and the VD may improve apolipoprotein levels, particularly in individuals at high CVD risk [11, 12]. However, to date, evidence regarding the effects of the MD and VD on a broad panel of apolipoproteins, especially in healthy adults at low-moderate risk, remains limited. The aim of this pilot work was to compare the effects of two diets universally recognized as healthy, such as the MD and the VD, on apolipoprotein levels and their relationship with traditional CVD risk factors such as lipid profile and inflammatory profile in participants with low-to-moderate CVD risk.

## Materials and methods

### Study population

Data presented in this paper were obtained from 52 clinically healthy participants at low-moderate risk (39 women; 13 men, with a mean age 49.1 ± 12.4 years) enrolled within the CARDIVEG (Cardiovascular Prevention with Vegetarian Diet) study, a trial conducted with the aim of comparing the effects of VD and MD on several cardiovascular disease (CVD) risk factors [10]. The study protocol and the general characteristics of the participants were extensively described in [13] and briefly summarized here. Participants with a low-to-moderate cardiovascular risk profile (< 5% at 10 years, according to the guidelines for CVD prevention of the European Society of Cardiology) [14] were recruited from the Clinical Nutrition Unit of Careggi University Hospital, Florence, Italy. Eligibility criteria included being overweight or obese (body mass index [BMI] ≥ 25 kg/m^2^) and the concomitant presence of at least one of the following CVD risk factors: total cholesterol levels > 190 mg/dL; LDL-cholesterol levels > 115 mg/dL; triglyceride levels > 150 mg/dL; fasting plasma glucose levels between 110 and 125 mg/dL. Ineligibility criteria were the presence of serious illness or unstable conditions, taking medication for any reason, being pregnant or lactating, the exclusion of meat, meat products, poultry, or fish from the diet in the past 6 months, or the participation in a weight loss program in the past 6 months.

### Study design

The study was a randomised, open, crossover clinical trial with two dietary intervention periods, each lasting 3 months. The start of the study was preceded by a 2-week run-in period necessary to assess the motivation and availability of the participants and to obtain a 3-day dietary record (two weekdays and one weekend day), which was analysed by a dietician using a nutrition-specific database. The participants enrolled were randomly assigned to a MD (n = 27) or a VD (n = 25) group as a first dietary intervention and then crossed over to the other dietary treatment. All participants were instructed not to alter their lifestyle or physical activity grade during the study, and no weight loss goal was given. Written informed consent was obtained from each participant. Clinical evaluations were performed at the baseline before the start of treatment, 3 months after the start of the first dietary intervention (at the time of crossing over) and then 3 months after the start of the second dietary intervention. The primary outcomes of the study were differences in changes in body weight, BMI, and fat mass from the baseline. The secondary outcomes were differences in changes on circulating cardiovascular risk markers and apolipoprotein levels from the baseline.

The study was approved by the Ethics Committee of the Tuscany Region, Careggi University Hospital (SPE 15.054) and registered at clinicaltrials.gov (identifier: NCT02641834), and adhered to the principles of the Declaration of Helsinki and the Data Protection Act.

### Dietary interventions and compliance

VD and MD were isocaloric between them, but hypocaloric with respect to the energy requirements of the participants. Both diets consisted of approximately 50–55% of energy from carbohydrate, 15–20% from protein and 25–30% from total fat (≤ 7% of energy from saturated fat, < 300 mg of cholesterol). The VD consisted of plant-based foods, eggs, milk and dairy products but completely excluded meat and meat products, poultry, fish and seafood. The MD was characterized by the consumption of all the food groups, including meat and meat products, poultry, and fish, although red meat consumption was limited to once a week. All participants were provided with a detailed 1-week menu plan, precise information on the foods that could be included or excluded, and a lot of different recipes for meal preparation. The dietary profiles for both VD and MD were calculated based on the portion sizes recommended by the Italian Recommended Dietary Allowances [15]. There was no difference between the two diets in the frequency of weekly servings of fruit and vegetables, cereals and olive oil. However, a higher frequency of weekly consumption of dairy products (21.5 vs. 18.5 servings), legumes (5 vs. 2.5), eggs (2 vs. 1), and nuts (2 vs. 1) was present in VD than in MD.

The adherence to the VD was assessed using a modified version of the National Health and Nutrition Examination Survey food questionnaire [16] and through unannounced telephone calls to participants, during which a 24-h diet recall interview was conducted. Participants were considered adherent to the VD if they reported no consumption of any animal flesh both in the questionnaire and in the interview. The adherence to MD was evaluated through the Medi-Lite validated questionnaire [17], considering participants who reported ≥ 10 points (in a scale ranging from 0 to 18) as adherents.

### Data collection

Data collection was carried out at the Clinical Nutrition Unit of Careggi University Hospital by standardised methods. All participants, who were required not to undertake strenuous physical activity during the day before the visit, were interviewed and examined between 6:30 a.m. and 9:30 a.m. after an overnight fasting period. Detailed information on demographics, risk factors, comorbidities, dietary and lifestyle habits was collected from each participant at the baseline. BMI, body composition and blood samples were obtained both at the beginning and at the end of the VD and MD intervention period. BMI was calculated as the weight (kg)/height (m^2^), with weight and height measured using a stadiometer. Body composition was determined by a bioelectrical impedance analysis device (TANITA, Arlington Heights, IL, model TBF-410).

### Laboratory measurements

Peripheral venous blood samples were collected at the beginning and at end of both dietary intervention periods into evacuated plastic tubes (Vacutainer; Becton Dickinson, Plymouth, UK). Samples were centrifuged at 4,000 rpm for 15 min (4 °C), and then stored in aliquots at − 80 °C. Circulating levels of apolipoproteins (ApoA-I, ApoB-100, ApoC-I, ApoC-III, ApoD, ApoE) were measured by Multiplex Luminex Assay using a custom kit (Bio-Plex Pro™ Human Apolipoprotein 10-Plex Assay, Bio-Rad), performed according to the manufacturer’s recommended protocol. Pro-and anti-inflammatory cytokines (interleukin (IL)-1ra, IL-4, IL-6, IL-8, IL-10, IL-12, IL-17, MCP-1, MIP-1β, VEGF, TNF-α, IP-10, IFN-γ) were determined by a Bio-Plex cytokine assay (Bio-Rad Laboratories Inc) according to the manufacturer’s instructions.

### Statistical analysis

Statistical analysis presented in this paper was performed using the statistical package IBM® SPSS® Statistics for Macintosh, version 28.0 (IBM Corp., Armonk, N.Y., USA). The results were reported as means ± standard deviations (SD), frequencies and percentages, or geometric means and 95% confidence intervals (CI), as appropriate. All data were treated as paired samples from a crossover study. MD and VD interventions were analysed combining the results obtained in the two intervention periods of both groups. Differences between the two groups at the baseline were evaluated using the Mann–Whitney U test. The χ^2^ test was used for dichotomous variables. A Shapiro–Wilk test was performed in order to check if data were normally distributed. To evaluate the effects of the VD and MD a general linear model for repeated measurements, adjusted for order of treatment and weight change was conducted. Because these analyses assume normal data distribution, non-distributed data were logarithmically transformed and further analyses were carried out with the processed data. However, to facilitate interpretation, the log data were reconverted to the original antilogarithmic scale and presented here as geometric means and 95% confidence intervals (CI). The possible dietary carryover effect, that is, the effect that considers whether the impact of the first treatment is still present when the patient enters the second treatment period, was analyzed. We evaluated the sequence effect, which considers whether the impact of VD and MD was different when the order of administration changed. This effect was estimated by comparing the geometric mean change difference between treatments in the VD group and in the MD group, after adjustment for order of treatment. Subgroup analyses were performed to evaluate possible differences in the changes of circulating apolipoprotein levels according to some characteristics of the study population, such as sex (women and men), age (less than or equal to 50 year-old and older than 50 years-old) or cardiovascular risk factors (total cholesterol levels > 190 mg/dL; LDL-cholesterol levels > 115 mg/dL; triglyceride levels > 150 mg/dL; fasting plasma glucose levels between 110 and 125 mg/dL). A Spearman’s correlation analysis was conducted to evaluate the relationship between changes in circulating apolipoprotein levels and changes in circulating lipid profile, inflammatory profile and dietary composition. Finally, a linear regression analysis adjusted for order of treatment and weight change was conducted in order to evaluate the relationship between changes in circulating apolipoprotein levels and changes in circulating lipid profile, inflammatory profile and dietary composition. P values < 0.05 were considered statistically significant.

## Results

### Baseline characteristics of the study population

Baseline characteristics of the study population according to the first dietary intervention are reported in Table 1. No significant differences were observed between the two groups in terms of demographics, anthropometrics, dietary habits, cardiovascular risk factors, lipid profile, inflammatory profile, and apolipoprotein levels at baseline.

**Table 1:** Baseline characteristics of the study population according to the first randomization

	All (n = 52)	MD (n = 27)	VD (n = 25)	*p* value
Demographics and anthropometrics				
Age, years	49.13 ± 12.40	48.11 ± 13.43	50.24 ± 11.37	*0.542*
Women, n (%)	39 (75)	20 (74.1)	19 (76)	*0.873*
Body weight, kg	82.77 ± 17.21	82.14 ± 15.93	83.45 ± 18.80	*0.787*
BMI, kg/m^2^	29.88 ± 4.79	29.54 ± 4.07	30.25 ± 5.53	*0.600*
Fat mass, kg	30.84 ± 11.67	30.01 ± 9.94	31.74 ± 13.44	*0.598*
Fat-free mass, kg	51.93 ± 10.95	52.13 ± 11.44	51.71 ± 10.62	*0.891*
Dietary profile				
Total energy, kcal/die	2,161 ± 590	2,048 ± 574	2284 ± 594	*0.117*
Carbohydrate, % of energy	46.68 ± 8.96	45.95 ± 9.91	47.46 ± 7.93	*0.848*
Protein, % of energy	17.30 ± 4.51	18.01 ± 3.82	16.53 ± 5.12	*0.085*
Total fat, % of energy	37.14 ± 7.11	37.28 ± 8.43	36.99 ± 5.52	*0.927*
Saturated fat, % of energy	7.94 ± 3.00	8.22 ± 3.52	7.64 ± 2.35	*0.721*
Dietary cholesterol, mg/die	204.73 ± 120.97	209.43 ± 142.68	199.65 ± 94.82	*0.934*
Dietary fiber, g/die	31.86 ± 53.46	24.19 ± 24.40	40.15 ± 72.74	*0.097*
Risk factors				
Current smokers, n (%)	8 (15.4)	6 (22.2)	2 (8)	*0.156*
Absent or light physical activity, n (%)	43 (82.7)	22 (81.5)	21 (84)	*0.810*
Triglycerides > 150 mg/dL, n (%)	14 (26.9)	6 (22.2)	8 (32)	*0.427*
Total cholesterol > 190 mg/dL, n (%)	38 (73.1)	19 (70.4)	19 (76)	*0.647*
LDL-cholesterol > 115 mg/dL, n (%)	36 (69.2)	19 (70.4)	17 (68)	*0.853*
Lipid profile				
Triglycerides, mg/dL	120.37 ± 64.31	111.85 ± 60.79	129.56 ± 67.94	*0.326*
Total cholesterol, mg/dL	211.73 ± 37.16	209.37 ± 39.96	214.28 ± 34.51	*0.639*
LDL-cholesterol, mg/dL	130,00 ± 34.26	126.52 ± 34.95	133.8 ± 33.8	*0.451*
HDL-cholesterol, mg/dL	57.65 ± 14.93	60.48 ± 15.97	54.60 ± 13.37	*0.158*
Inflammatory profile				
IL-1ra, pg/mL	27.34 ± 19.35	30.74 ± 20.23	23.67 ± 18.04	*0.128*
IL-4, pg/mL	0.26 ± 0.33	0.21 ± 0.34	0.31 ± 0.33	*0.268*
IL-6, pg/mL	1.55 ± 1.42	1.59 ± 1.41	1.51 ± 1.45	*0.776*
IL-8, pg/mL	6.29 ± 5.72	5.92 ± 5.07	6.68 ± 6.43	*0.847*
IL-10, pg/mL	5.68 ± 7.70	5.70 ± 8.04	5.66 ± 7.46	*0.920*
IL-12, pg/mL	30.46 ± 20.06	32.83 ± 18.45	27.89 ± 21.75	*0.170*
IL-17, pg/mL	10.23 ± 12.46	12.32 ± 15.43	7.97 ± 7.86	*0.241*
MCP-1, pg/mL	38.41 ± 22.93	41.29 ± 23.15	35.31 ± 22.74	*0.145*
MIP-1β, pg/mL	67.23 ± 31.92	64.87 ± 32.46	69.77 ± 31.78	*0.527*
VEGF, pg/mL	79.94 ± 51.08	89.12 ± 49.00	70.02 ± 52.40	*0.109*
TNF-α, pg/mL	5.37 ± 5.76	4.28 ± 3.96	6.55 ± 7.12	*0.374*
IP-10, pg/mL	629.67 ± 549.71	576.57 ± 368.58	687.01 ± 698.83	*0.963*
IFN-γ, pg/mL	7.39 ± 10.87	4.41 ± 5.15	10.61 ± 14.20	*0.166*
Apolipoproteins				
ApoA-I, mg/dL	166.52 ± 53.20	172.78 ± 54.96	159.77 ± 51.48	*0.384*
ApoB, mg/dL	78.98 ± 17.09	75.54 ± 15.48	82.69 ± 18.25	*0.133*
ApoB/ApoA-I	0.52 ± 0.18	0.48 ± 0.17	0.56 ± 0.19	*0.106*
ApoC-I, mg/dL	29.14 ± 17.13	32.91 ± 19.39	25.07 ± 13.53	*0.100*
ApoC-III, mg/dL	6.45 ± 2.43	6.62 ± 2.81	6.25 ± 1.97	*0.586*
ApoD, μg/dL	60.28 ± 25.93	64.12 ± 30.33	56.13 ± 19.92	*0.271*
ApoE, mg/dL	2.77 ± 1.51	2.83 ± 1.52	2.71 ± 1.52	*0.785*

Bold values used to highlight significant differences

Italics have been used to report the *p*-value

*Apo* apolipoprotein, *BMI* Body Mass Index, *HDL* high-density lipoprotein, *IFN* interferon, *IL* interleukin, *IP* interferon-γ–induced protein, *LDL* low-density lipoprotein, *MCP* monocyte chemoattractant protein, *MD* Mediterranean diet, *MIP* macrophage inflammatory protein, *TNF* tumor necrosis factor, *VEGF* vascular endothelial growth factor, *VD* Vegetarian diet. Data are reported as mean ± SD or number and percentage as appropriate

### Changes after dietary interventions

#### Dietary intakes and anthropometric parameters

Dietary interventions with the two proposed diets determined significant changes in dietary intakes (Additional file 1: Table S1). A significant decrease of total energy, total fats, and dietary cholesterol and an increase in total carbohydrates was observed in both diets, while VD resulted in a significant reduction in protein and an increase in dietary fibre to a greater extent than MD. Changes in anthropometric parameters after the two interventions are reported in Table 2. Both MD and VD resulted in significant reductions in body weight, BMI and fat mass, with VD that led to a significant reduction also in fat-free mass.

**Table 2:** Changes in anthropometric parameters, lipid profile, inflammatory profile and apolipoproteins after 3 months of dietary interventions

	MD pre (n = 52)	MD post (n = 52)	Δ change	*p* value	VD pre (n = 52)	VD post (n = 52)	Δ change	*p* value	*p*_*Δ*_
Anthropometric parameters									
Body weight, kg	80.94 (76.36;85.52)	78.78 (74.35;83.22)	− 2.16 (− 2.89;− 1.42)	** < *0.001***	80.88 (76.18; 85.58)	78.97 (74.57; 83.38)	− 1.90 (− 2.75;− 1.05)	** < *0.001***	*0.361*
BMI, kg/m^2^	29.22 (27.98; 30.45)	28.44 (27.25; 29.64)	− 0.77 (− 1.02;− 0.52)	** < *0.001***	29.22 (27.89; 30.55)	28.55 (27.31; 29.79)	− 0.67 (− 0.98;− 0.36)	** < *0.001***	*0.263*
Fat mass, kg	29.69 (26.47; 32.91)	27.94 (24.94; 30.95)	− 1.75 (− 2.38;− 1.12)	** < *0.001***	29.37 (26.16; 32.58)	28.20 (24.94; 31.47)	− 1.17 (− 1.85;− 0.48)	***0.001***	*0.160*
Free fat mass, kg	51.25 (48.28; 54.21)	50.84 (47.87; 53.81)	− 0.41 (− 0.85; 0.04)	*0.074*	51.50 (48.47; 54.54)	50.77 (47.90; 53.64)	− 0.74 (− 1.37;− 0.11)	***0.022***	*0.385*
Lipid profile									
Triglycerides, mg/dL	123.01 (103.33; 142.83)	112.02 (95.58; 128.46)	− 11.06 (− 20.11;− 2.01)	***0.018***	115.58 (99.28; 131.87)	125.23 (104.42; 146.05)	9.65 (− 4.55; 23.86)	*0.178*	***0.007***
Total cholesterol, mg/dL	203.54 (193,51; 213.57)	203.65 (194.94; 212.37)	0.12 (− 6.41; 6.64)	*0.972*	207.27 (197.84; 216.70)	202.21 (192.48; 211.94)	− 5.06 (− 11.40; 1.29)	*0.116*	*0.598*
LDL-cholesterol, mg/dL	123.56 (113.93; 133.19)	125.29 (117.09; 133.49)	1.73 (− 4.40; 7.86)	*0.573*	128.48 (120.01; 136.95)	122.05 (112.73; 131.36)	− 6.44 (− 12.48;− 0.39)	***0.038***	*0.324*
HDL-cholesterol, mg/dL	55.37 (51.35; 59.38)	55.96 (52.32; 59.60)	0.60 (− 1.47; 2.66)	*0.565*	55.67 (52.24; 59.11)	54.96 (51.08; 58.84)	− 0.71 (− 2.60; 1.18)	*0.452*	*0.157*
Inflammatory profile									
IL-1ra, pg/mL	23.60 (18.15; 29.05)	15.73 (11.37; 20.08)	− 7.87 (− 12.82;− 2.92)	***0.002***	18.05 (13.55; 22.56)	14.93 (10.53; 19.32)	− 3.13 (− 7.74; 1.48)	*0.179*	*0.315*
IL-4, pg/mL	0.25 (0.16; 0.35)	0.29 (0.20; 0.37)	0.03 (− 0.5; 0.1)	*0.407*	0.23 (0.15; 0.31)	0.29 (0.20; 0.38)	0.06 (− 0.00; 0.12)	*0.067*	*0.823*
IL-6, pg/mL	1.42 (1.09; 1.75)	1.09 (0.82; 1.35)	− 0.33 (− 0.56;− 0.10)	***0.005***	1.31 (0.97; 1.65)	1.06 (0.83; 1.28)	− 0.26 (− 0.55; 0.04)	*0.083*	*0.445*
IL-8, pg/mL	7.07 (4.86; 9.27)	6.18 (3.97; 8.38)	− 0.89 (− 3.76; 1.98)	*0.536*	8.30 (5.78; 10.82)	5.16 (3.19; 7.12)	− 3.14 (− 6.15;− 0.12)	***0.042***	*0.189*
IL-10, pg/mL	5.88 (3.46; 8.30)	3.69 (2.08; 5.30)	− 2.19 (− 3.81;− 0.58)	***0.009***	4.80 (2.88; 6.72)	4.99 (2.72; 7.26)	0.19 (− 1.56; 1.94)	*0.827*	***0.046***
IL-12, pg/mL	25.45 (19.99; 30.91)	18.18 (13.22; 23.14)	− 7.28 (− 11.35;− 3.20)	** < *0.001***	21.28 (16.58; 25.99)	18.27 (13.67; 22.87)	− 3.01 (− 6.12; 0.09)	*0.057*	*0.083*
IL-17, pg/mL	9.22 (5.95; 12.49)	4.69 (3.05; 6.32)	− 4.53 (− 7.37;− 1.70)	***0.002***	6.54 (4.44; 8.65)	5.75 (4.36; 7.13)	− 0.80 (− 2.75; 1.16)	*0.416*	***0.012***
MCP-1, pg/mL	32.95 (27.41; 38.49)	18.75 (16.24; 21.27)	− 14.20 (− 19.53;− 8.87)	** < *0.001***	27.92 (23.06; 32.78)	21.53 (17.79; 25.27)	− 6.39 (− 10.78;− 2.01)	***0.005***	*0.059*
MIP-1β, pg/mL	59.88 (52.06; 67.70)	48.94 (41.72; 56.16)	− 10.94 (− 18.85;− 3.02)	***0.008***	58.63 (49.99; 67.27)	47.18 (41.06; 53.29)	− 11.45 (− 20.18;− 2.72)	***0.011***	*0.621*
VEGF, pg/mL	68.55 (56.10; 80.99)	49.06 (39.90; 58.23)	− 19.48 (− 28.97;− 9.99)	** < *0.001***	58.97 (47.47; 70.47)	46.21 (36.55; 55.86)	− 12.76 (− 20.61;− 4.90)	***0.002***	*0.706*
TNF-α, pg/mL	4.61 (3.40; 5.82)	4.32 (3.01; 5.63)	− 0.29 (− 1.42; 0.84)	*0.608*	5.06 (3.55; 6.58)	4.38 (3.41; 5.35)	− 0.68 (− 1.91; 0.54)	*0.267*	*0.907*
IP-10, pg/mL	566.44 (439.57; 693.31)	501.80 (400.57; 603.04)	− 64.64 (− 146.45; 17.18)	*0.119*	609.78 (462.31; 757.24)	491.37 (377.37; 605.37)	− 118.41 (− 211.20;− 25.61)	***0.013***	*0.168*
IFN-γ, pg/mL	5.16 (3.55; 6.77)	6.02 (4.02; 8.01)	0.86 (− 1.05; 2.77)	*0.371*	7.89 (4.84; 10.95)	4.98 (3.62; 6.35)	− 2.91 (− 5.26;− 0.56)	***0.016***	*0.368*
Apolipoproteins									
ApoA-I, mg/dL	158.31 (145.01; 171.62)	167.91 (154.46; 181.36)	9.60 (− 9.17; 28.36)	*0.309*	158.14 (143.50; 172.78)	162.37 (149.99; 174.75)	4.23 (− 14.37; 22.83)	*0.649*	*0.626*
ApoB, mg/dL	78.02 (73.08; 82.96)	76.78 (71.58; 81.99)	− 1.24 (− 7.36; 4.88)	*0.686*	78.60 (73.43; 83.78)	79.14 (73.66; 84.62)	0.54 (− 5.52; 6.60)	*0.859*	*0.552*
ApoB/ApoA-I	0.54 (0.48; 0.59)	0.50 (0.45; 0.55)	− 0.04 (− 0.11; 0.03)	*0.213*	0.54 (0.49; 0.60)	0.53 (0.48; 0.59)	− 0.01 (− 0.07; 0.05)	*0.726*	*0.435*
ApoC-I, mg/dL	32.01 (26.56; 37.46)	35.39 (29.32; 41.45)	3.38 (− 3.95; 10.70)	*0.358*	30.76 (25.71; 35.80)	38.28 (31.79; 44.77)	7.52 (1.22; 13.83)	***0.020***	*0.265*
ApoC-III, mg/dL	6.47 (5.73; 7.22)	6.60 (5.87; 7.32)	0.12 (− 0.79; 1.03)	*0.791*	6.60 (5.95; 7.25)	6.23 (5.48; 6.98)	− 0.37 (− 1.13; 0.38)	*0.326*	*0.483*
ApoD, μg/dL	59.76 (51.91; 67.61)	63.48 (56.49; 70.47)	3.72 (− 5.47; 12.91)	*0.420*	60.89 (54.37; 67.40)	64.82 (56.45; 73.20)	3.94 (− 3.05; 10.93)	*0.263*	*0.868*
ApoE, mg/dL	3.05 (2.62; 3.47)	2.99 (2.62; 3.37)	− 0.05 (− 0.54; 0.44)	*0.838*	2.97 (2.59; 3.36)	3.40 (2.99; 3.81)	0.42 (− 0.01; 0.856)	*0.054*	*0.138*

Bold values used to highlight significant differences

Italics have been used to report the *p*-value

*Apo* apolipoprotein, *BMI* Body Mass Index, *HDL* high-density lipoprotein, *IFN interferon, , IL* interleukin, *IP* interferon-γ–induced protein, *LDL* low-density lipoprotein, *MCP* monocyte chemoattractant protein, *MD* Mediterranean diet, *MIP* macrophage inflammatory protein, *TNF* tumor necrosis factor, *VEGF* vascular endothelial growth factor, *VD* vegetarian diet. Data are reported as mean ± SD or number and percentage as appropriate

Data are reported as geometric mean and 95% confidence intervals. General linear model for repeated measurements adjusted for order of treatment (anthropometric parameters, lipid profile, inflammatory profile and apolipoproteins) and weight change (lipid profile, inflammatory profile and apolipoproteins)

#### Lipid profile and inflammatory profile

Regarding the lipid profile, VD led to a significant reduction in LDL cholesterol (− 5%; *p* = 0.038), while MD led to a significant reduction in plasma triglycerides (− 9%; *p* = 0.018) with a significant difference between the two interventions (*p* = 0.007) (Table 2). In addition, both MD and VD led to a reduction in most of the inflammatory parameters analysed, with a statistically significant difference between the two diets for IL-10 and IL-17, which significantly decreased only after MD (− 37.2%; *p* = 0.009 and − 49.1%; *p* = 0.002, respectively) (Table 2).

#### Apolipoproteins

As for apolipoproteins, a statistically significant change was observed only for ApoC-I after VD (+ 24.4%; *p* = 0.020). A similar increasing trend was observed after MD and VD for ApoA-I (+ 6.1% for MD and + 2.7% for VD), ApoC-I (+ 10.6% for MD and + 24.4% for VD) and ApoD (+ 6.2% for MD and + 6.5% for VD). In addition, after following the MD and the VD, the ApoB/ApoA-I decreased by 7.4% and 1.9% respectively. On the other hand, an opposite trend was observed for ApoB (− 1.6% for MD and + 0.7% for VD), ApoC-III (+ 1.8% for MD; − 5.6% for VD) and ApoE (− 1.6% for MD and + 14.1% for VD).

### Correlation analysis for apolipoproteins

**Fig. 1:**
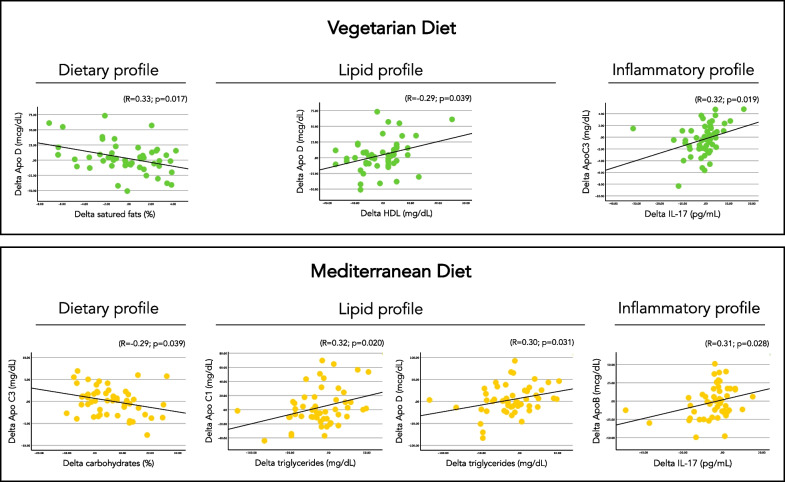
Association between apolipoproteins and nutrients intake and lipid profile after the two diets

After intervention with MD, a statistically significant negative correlation emerged between ApoC-III and percentage of carbohydrates (R = − 0.29; *p* = 0.039) whereas after the intervention with VD a statistically significant negative correlation emerged between ApoD and percentage of saturated fats (R = − 0.38; *p* = 0.006) (Fig. 1). By analysing correlations between apolipoproteins and lipid variables, a statistically significant positive correlation emerged after the intervention with MD between the change in plasma triglycerides and the change in ApoC-I (R = 0.32; *p* = 0.020) and ApoD (R = 0.30; *p* = 0.031) levels. On the other hand, after the VD intervention a significant positive correlation emerged between change in HDL levels and ApoD (R = 0.33; *p* = 0.017) (Fig. 1). Finally, change in IL-17 resulted to be positively correlated with change in ApoB after MD (R = 0.31; *p* = 0.028) and with change in ApoC-III after VD (R = 0.32; *p* = 0.019). The same results were also confirmed by linear regression analysis adjusted for possible confounders (i.e. order of treatment and weight change).

### Subgroup analyses for apolipoproteins

**Fig. 2:**
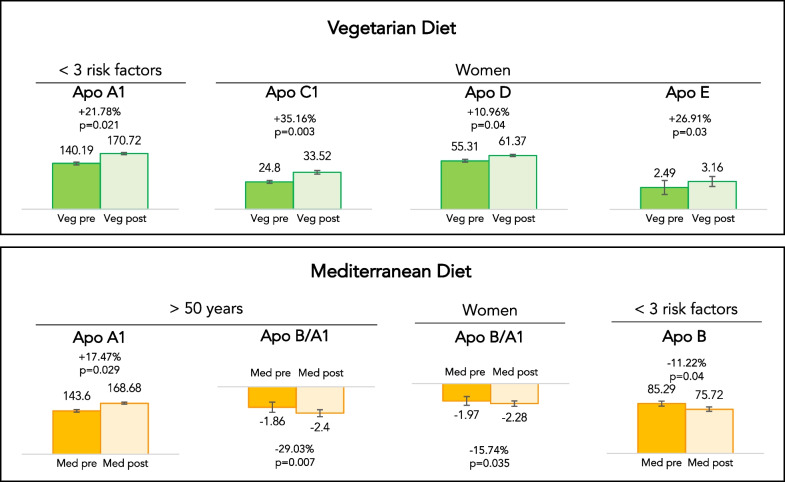
Changes in apolipoprotein levels according to sex, age and cardiovascular risk factors after 3 months of dietary interventions with VD and MD

Subgroup analyses were conducted according to sex, age, and cardiovascular risk factors. Women showed some significant changes after both dietary interventions; in fact, VD led to a significant increase of ApoC-I (+ 35.2%; *p* = 0.003), ApoD (+ 11%; *p* = 0.04) and ApoE (+ 26.9%; *p* = 0.03), while MD led to a significant decrease of ApoB/ApoA-I (− 15.7%; *p* = 0.035). Regarding age, MD led to an increase of ApoA-I (+ 17.5%; *p* = 0.029) and to a decrease of ApoB/ApoA-I (− 29%; *p* = 0.007) in people older than 50 years-old. Finally, people with less than 3 cardiovascular risk factors experienced a statistically significant increase of ApoA-I (+ 21.8%; *p* = 0.021) after VD and a decrease of ApoB (− 11.2%; *p* = 0.04) after MD (Fig. 2).

## Discussion

The present is the first clinical trial comparing the effects of a 3-month dietary intervention with MD and VD on apolipoprotein levels and their relationship with traditional CV risk factors such as lipid and inflammatory profile in healthy adults at low-moderate risk. In our study, several changes of apolipoproteins were reported after both MD and VD, with statistically significant increase of ApoC-I levels only after VD. The benefits of both diets were greater in certain population subgroups, such as women, individuals older than 50 years-old, and those with < 3 cardiovascular risk factors, especially after the Mediterranean-type diet that improved levels of ApoA-I, ApoB and ApoB/ApoA-I ratio in these subgroups of participants.

CVD remains the leading cause of death worldwide, so scientific interest has focused on investigating possible modifiable factors to attenuate this risk. Recent guidelines of the European Society of Cardiology highlight the importance of ApoB as a marker of cardiovascular risk, given its presence in lipoproteins associated with higher risk, such as VLDL, IDL and LDL [3]. Moreover, several studies suggest a protective role for ApoA-I, as it is found mainly in HDL lipoproteins and plays a role in reverse cholesterol transport. Both are used to calculate the ApoB/ApoA-I ratio, which has been suggested as a better risk marker than total cholesterol/HDL cholesterol ratio [2, 18]. However, to date, evidence regarding the effects of healthy dietary patterns such as MD and VD on apolipoproteins remains limited. In our study, after 3 months of intervention, MD determined significant improvement in the subgroup of women and people older than 50 years who reported a decrease in ApoB/ApoA-I ratio, and an increase in ApoA-I in those older than 50 years. These data agree with those published by Solà et al. [12] who reported an improvement in ApoA-I levels and the ApoB/ApoA-I ratio, as well as a significant reduction in ApoB levels after 3 months of MD in adults aged 55 to 80 years at high cardiovascular risk. As for VD, no improvement in ApoB levels was observed, despite a recent meta-analysis that found a 10% reduction in LDL levels together with a 14% reduction in ApoB values after a vegetarian or vegan diet [11], with differences explainable by the heterogeneity of the diets administered. On the other hand, VD determined a significant increase of ApoC-I levels, and this could be related to the trend toward increased triglyceride levels, since it is an apolipoprotein involved in the metabolism of triglyceride-rich lipoproteins.

In this study, we showed different associations between apolipoprotein levels and specific nutrients such as carbohydrates and saturated fats. An unexpected result of our study was the inverse association between changes in carbohydrate intake and ApoC-III after MD. ApoC-III is a crucial player in triglyceride-rich lipoprotein metabolism, influencing vascular biology and atherosclerosis through several mechanisms [19]. In fact, different studies show that higher levels of ApoC-III are related to high levels of triglycerides. Due to the relationship between high carbohydrate intake and increased plasma triglyceride levels [20] a positive association between changes of both factors would be expected, as otherwise observed by Hieronimus et al. [21]. In our study, instead, we observed an inverse association between changes of carbohydrates and ApoC-III. This could be explained by the fact that MD is characterized by a high intake of vegetables, fruits and whole grains that are a source of complex and non-refined carbohydrates, so suggesting that probably not only the amount of carbohydrates but also the type of it can influence ApoC-III levels.

Regarding inflammatory parameters, we found a significant difference between the two diets for IL-10 and IL-17, which decreased only after the MD intervention. Although different studies have evaluated the possible relationship between both dietary profiles and inflammatory parameters [22, 23], to the best of our knowledge, this is the first study to evaluate the possible correlations of apolipoproteins and inflammatory profile after 3-month dietary intervention with MD and VD. We observed that levels of pro-inflammatory IL-17 correlated positively with levels of apolipoproteins associated with increased CVD risk [3, 25], in particular with ApoB after MD and with ApoC-III change after VD. In line with the present study, the NU-AGE study found that MD intervention was associated with positive modulation of gut microbiota which was inversely related to IL-17 levels [24], thus suggesting that MD can modulate the inflammatory response through different mechanisms.

The present study, despite some limitations as the limited number of participants enrolled – most of them women –, the lack of a washout period, the dietary interventions limited to only 3 months, as well as the fact that these results cannot be extended to other populations, presents some strengths such as the interventional design with two diets similar in terms of total caloric intake and nutritional composition, as well as the high rate of adherence of the participants to the diets. Indeed, the cross-over design allowed us to compare MD and VD in the same group of participants, minimizing interindividual variability, since the participants acted as their own control. In addition, no participants during the study were taking drugs or medication to treat dyslipidemia, which could have influenced the results and, finally, the study included the analysis of a broad panel of apolipoproteins and their possible relationship between two dietary profiles and their nutrients and several lipid and inflammatory parameters.

## Conclusions

To conclude, healthy dietary patterns such as VD and MD both seem to improve the overall CVD risk of clinically healthy participants at low-moderate risk by modulating the lipid and inflammatory profile. MD appears to have a greater positive effect on apolipoprotein levels, especially in some subgroups such as women or individuals older than 50 years-old or < 3 CVD risk factors. Thus, our results confirm how a healthy at low-moderate risk, balanced diet is critical for reducing CVD risk and suggest how diet can improve, even in the short term, novel potential markers of CVD risk.

### Supplementary Information

**Additional file 1: Table S1.** Variations in dietary intake according to dietary intervention.

## Data Availability

Additional data are available from the corresponding author on reasonable request.
