# Supplementary material for: Effects of a dietary intervention with lacto-ovo-vegetarian and Mediterranean diets on apolipoproteins and inflammatory cytokines: results from the CARDIVEG study
Source: Nutr Metab (Lond). 2024 Feb 1;21:9. doi: 10.1186/s12986-023-00773-w (PMC10832112; doi:10.1186/s12986-023-00773-w)
Supplement: Supplementary file 1 — Additional file 1: Table S1. Variations in dietary intake according to dietary intervention. [file 12986_2023_773_MOESM1_ESM.docx]

**Supplementary material**

**Supplementary Table S1.** Variations in dietary intake according to dietary intervention

|  | **MD (n = 52)** | **VD (n = 52)** | ***p value*** |
| --- | --- | --- | --- |
| **Total energy,** kcal/die | -632.20 ± 537.67 * | -646.36 ± 537.81 * | *0.810* |
| **Carbohydrates,** % of energy | 5.63 ± 9.01 * | 7.48 ± 8.96 * | *0.288* |
| **Proteins,** % of energy | 1.31 ± 4.53 * | -1.86 ± 4.51 * | ***<0.001*** |
| **Total fats,** % of energy | -8.05 ± 7.11 * | -6.71 ± 7.11 * | *0.249* |
| **Saturated fats,** % of energy | -0.54 ± 3.00 | -0.42 ± 3.00 | *0.767* |
| **Dietary cholesterol,** mg/die | -50.04 ± 117.41 * | -111.90 ± 117.96 * | ***0.003*** |
| **Dietary fiber,** g/die | -2.01 ± 52.96 * | 4.63 ± 52.82 * | ***<0.001*** |

Data are reported as mean ± standard deviation.

MD: Mediterranean Diet; VD: lacto-ovo vegetarian Diet.

* denotes p<0.05 for change (after the intervention period vs before the intervention period)
